# Axillary Metastasis from an Occult Tubal Serous Carcinoma in a Patient with Ipsilateral Breast Carcinoma: A Potential Diagnostic Pitfall

**DOI:** 10.1155/2014/534034

**Published:** 2014-07-09

**Authors:** Chantal Atallah, Gulbeyaz Altinel, Lili Fu, Jocelyne Arseneau, Atilla Omeroglu

**Affiliations:** McGill University Health Center, Montreal, QC, Canada H3A 1A1

## Abstract

Axillary nodal metastasis from a nonmammary neoplasia is much rarer than diseases associated with a primary breast carcinoma. However, this has to be considered in the differential diagnosis of nodal disease in patients with a history of breast cancer. Here, we report the case of a 73-year-old female with a past medical history of breast cancer, presenting with an ipsilateral axillary metastatic carcinoma. The immunohistochemical profile of the metastatic lesion was consistent with a high grade serous carcinoma. After undergoing a total abdominal hysterectomy and salpingo-oophorectomy, thorough pathological examination revealed two microscopic foci of serous carcinoma in the right fallopian tube, not detectable by preoperative magnetic resonance imaging. In this context, the poorly differentiated appearance of the metastatic tumor and positive staining for estrogen receptor, might lead to a misdiagnosis of metastatic breast carcinoma. As the therapeutic implications differ, it is important for the pathologist to critically assess axillary lymph node metastases, even in patients with a past history of ipsilateral breast carcinoma and no other known primary tumors.

## 1. Introduction

Invasive breast carcinoma is the most common malignancy in women and the most common solid malignancy to metastasize to the axilla since about 50% of cases present with some degree of nodal disease [1, 2]. In breast carcinoma, late metastatic recurrences to the axilla have been reported, with the longest interval being up to 22 years after the original diagnosis [3]. On the other hand, less than 3% of metastases to the axilla originate from nonmammary sites, usually in the setting of widespread disease [4, 5]. An occult neoplasia first presenting as a nonmammary axillary metastasis is a much rarer event, and consequently the preemptive site of origin of these lesions is breast. In a recent series, the most common origin for nonmammary axillary metastasis was high grade serous carcinoma arising from the ovary, adding up to 11% of cases [5]. However, the features of breast and Müllerian tumors can overlap both morphologically and immunohistochemically, potentially leading to a diagnostic pitfall.

## 2. Results

**Figure 1:**
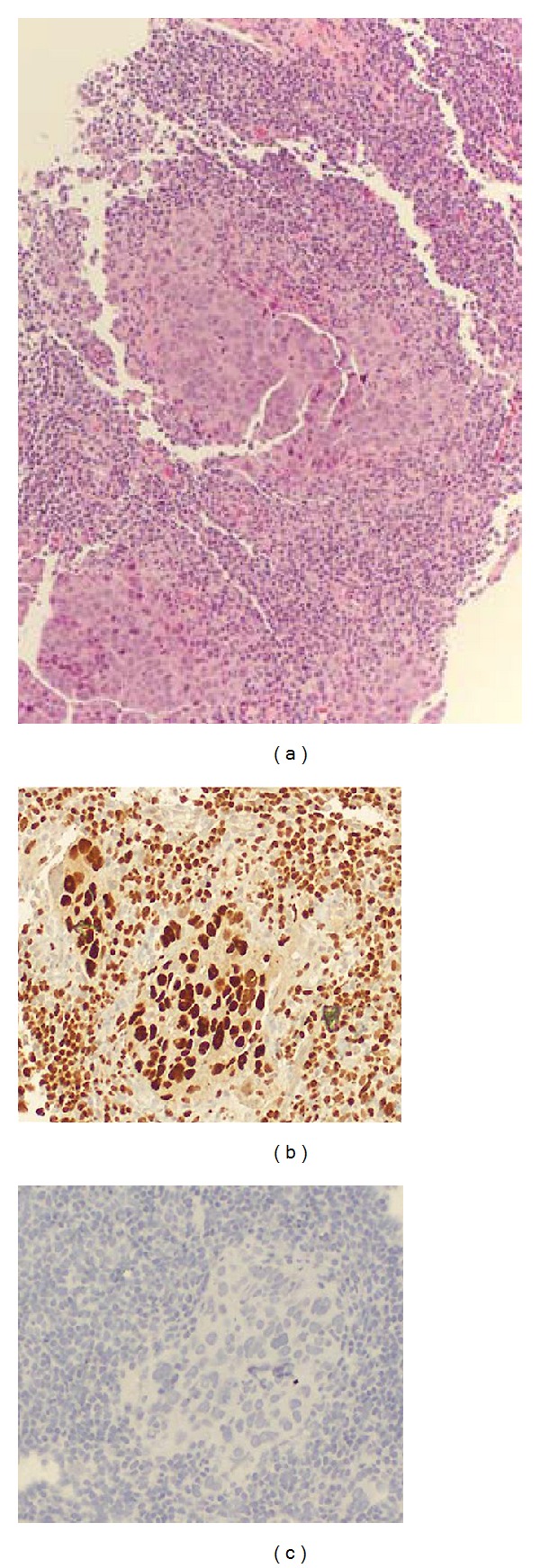
(a) H&E stained section from the biopsy core obtained from the axillary node. Poorly differentiated cells with a vague papillary configuration are seen. (b) Pax-8 staining performed on core biopsy shows nuclear staining. (c) Mammaglobin staining performed on core biopsy is negative.

**Figure 2:**
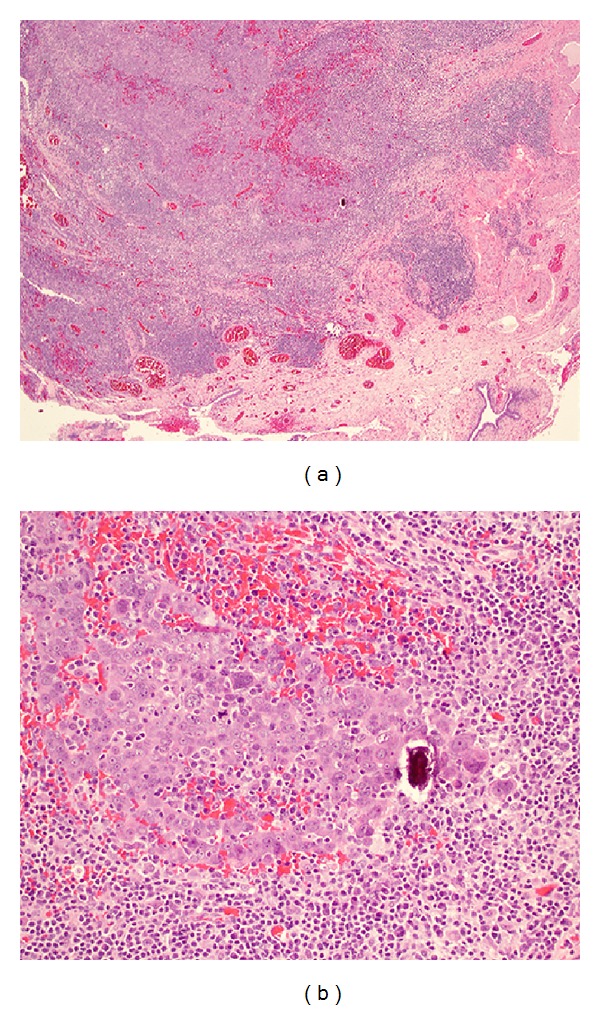
(a) H&E stained section, including the fimbriated end of the fallopian tube from the total abdominal hysterectomy and salpingo-oophorectomy specimen, demonstrates an invasive lesion. (b) H&E stained section, at a higher magnification, shows high grade serous carcinoma with psammomatous calcification, sharing the morphology of the axillary metastasis (Figure 1) and inguinal metastasis (not shown).

We report here the case of a 73-year-old woman with a past medical history of primary carcinoma in her right breast 16 years prior treated with mastectomy and partial axillary node dissection, who presented with enlarged right axillary lymph nodes, clinically consistent with recurrent breast carcinoma. The original primary tumor was not available for review. A core biopsy of the largest node was performed and histology showed a metastatic poorly differentiated carcinoma. An immunohistochemical panel was performed. The lesion was positive for PAX8, WT1, p53, p16, CK7, and ER, while it was negative for CK20, PR, Her2/neu, CDX-2, TTF1, and mammaglobin (Figure 1). Altogether, these results were consistent with the diagnosis of high grade serous carcinoma, likely of ovarian, tubal, or peritoneal origin. Subsequent axillary dissection for tumor debulking showed two metastatic lymph nodes with the largest measuring 3 cm across. The patient underwent magnetic resonance imaging (MRI) as part of her management which detected the presence of inguinal adenopathy, the largest measuring 2.5 cm across. The ovaries and tubes were unremarkable on imaging. She underwent a total abdominal hysterectomy with bilateral salpingo-oophorectomy (TAH-BSO). Examination of the pathological material showed two small foci of invasive high grade serous carcinoma located within the fimbriae of the right tube, each measuring less than 2 mm across (Figure 2). Serous tubal carcinoma in situ was also present. The largest inguinal lymph node was positive for small foci of tumoral cells with identical high grade morphology.

## 3. Discussion

Although the slides of the original primary breast carcinoma were not available for review and detailed immunohistochemistry, we considered the staining results of the tumor in the lymph node biopsy sufficiently convincing for the serous character and ovarian/tubal origin of the tumor. Metastasis to the axilla from nonmammary primary tumors constitutes less than 3% of all axillary metastases. The most common solid tumor is carcinoma (58%), followed by melanoma (22%) and sarcoma (20%) [6]. Among carcinomas, the most frequently encountered are lung, skin, stomach, and ovary [4]. In one recent series by DeLair et al. [5], serous ovarian carcinoma was the most common malignancy to metastasize to the breast or axilla, representing 11% of the cases. Morphologically, in that series, it was also the tumor most often misdiagnosed as a primary carcinoma of the breast. Primary tubal serous carcinoma shares the morphological features of ovarian carcinoma but is a much rarer tumor, with an incidence of 0.41 cases per 100,000. However, there is increasing evidence that in fact they are the same tumor, with ovarian lesions arising from the fallopian tube epithelium [7, 8]. The lymphatic drainage of the fallopian tube and ovaries follows four different pathways, which explains the location of metastasis. The two principal pathways are drainage of the proximal part of the tube by the para-aortic nodes and drainage of the distal part of the tube, near the fimbriae, by the pelvic nodes [9]. Additionally, disease may spread to the inguinal nodes by invading the vessels coursing by the round ligament which are drained by the inguinal nodes. Lastly, supradiaphragmatic spread is thought to occur through invasion of the central lymphatic duct which bypasses the para-aortic nodes [10]. Furthermore, as is illustrated in this case, serous tubal carcinoma has been recognized to metastasize at very early disease stages [9]. The great confounder in this case is the past history of ipsilateral breast carcinoma, which is also a unique feature of this case. High grade serous carcinoma, whether peritoneal, tubal, or ovarian, shares overlapping morphologic features with high grade invasive ductal carcinoma. Both tumors may exhibit a papillary, solid, or glandular growth pattern and may feature tumor necrosis. Furthermore, both tumors share similar immunostaining with ER, PR, and CK7, which makes a narrow panel of limited usefulness. PAX-8, which was positive in 87% of primary ovarian carcinomas in one study and 0% of primary breast carcinomas, is the stain of choice, followed by WT-1, which was found to be positive in 85% of primary ovarian carcinomas and 2% of primary breast carcinomas, with the exception of mucinous carcinomas, which were found to be positive in 64% of cases [5, 11–13]. Lastly, the possibility of serous adenocarcinoma arising from endosalpingiosis should be entertained if no primary tumor was found during thorough examination of the TAH-BSO specimen. Rare cases have been reported in the literature within peritoneal and inguinal locations [14, 15]. Immunohistochemistry would not be contributive in this case. Florid papillary endosalpingiosis of the axillary nodes has also been reported before [16]. Morphology—recognizing the absence of overt atypia—is crucial in establishing the correct diagnosis.

In conclusion, in cases like the one showcased here, the past clinical history acts as a confounder, especially with previous disease ipsilateral to the adenopathy. The relevant use of immunohistochemistry on the axillary core biopsy was crucial to reach the right diagnosis and to deliver appropriate patient care and management. Furthermore, a case of collision metastasis to the axilla by both breast and ovarian tumors has been reported, so diligent examination of each metastasis is warranted [17].
